# Refined soybean oil wastewater treatment and its utilization for lipid production by the oleaginous yeast *Trichosporon fermentans*

**DOI:** 10.1186/s13068-018-1306-6

**Published:** 2018-11-01

**Authors:** Dayu Yu, Xiaoning Wang, Xue Fan, Huimin Ren, Shuang Hu, Lei Wang, Yunfen Shi, Na Liu, Nan Qiao

**Affiliations:** 1 Sci-Tech Center for Clean Conversion and High-valued Utilization of Biomass, Jilin Province, Northeast Electric Power University, Jilin, 132012 China; 2 Key Laboratory of Groundwater Resources and Environment, Ministry of Education, Jilin University, Changchun, 130021 China; 3 School of Chemical Engineering, Northeast Electric Power University, Jilin, 132012 China; 4 School of Civil Engineering and Architecture, Northeast Electric Power University, Jilin, 132012 China

**Keywords:** Refined soybean oil wastewater, *Trichosporon fermentans*, Microbial oil, Wastewater treatment, Biodiesel, Chemical oxygen demand, Oil content

## Abstract

**Background:**

The release of refined soybean oil wastewater (RSOW) with a high chemical oxygen demand (COD) and oil content burdens the environment. The conversion of RSOW into lipids by oleaginous yeasts may be a good way to turn this waste into usable products.

**Results:**

The oleaginous yeast *Trichosporon fermentans* was used for treating the RSOW without sterilization, dilution, or nutrient supplementation. It was found that the COD and oil content of the RSOW were removed effectively; microbial oil was abundantly produced in 48 h; and the phospholipids in the RSOW tended to contribute to a higher biomass and microbial lipid content. With Plackett–Burman design and response surface design experiments, the optimal wastewater treatment conditions were determined: temperature 28.3 °C, amount of inoculum 5.9% (v/v), and initial pH 6.1. The optimized conditions were used in a 5-L bioreactor to treat the RSOW. The maximum COD degradation of 94.7% was obtained within 40 h, and the removal of the oil content was 89.9%. The biomass was 7.9 g/L, the lipid concentration was 3.4 g/L, and the lipid content was 43% (w/w). The microbial oil obtained, with a main component of unsaturated fatty acids, was similar to vegetable oils and was suggested as a potential raw material for biodiesel production.

**Conclusion:**

*Trichosporon fermentans* can be effectively used for RSOW treatment, and lipid production and can complete pretreatment and biochemical treatment simultaneously, allowing the utilization of RSOW, which both solves an environmental problem and positively impacts the use of resources. These results provide valuable information for developing and designing more efficient waste-into-lipid bioprocesses.

## Background

Soybean oil is one of the most widely used edible oils in the world. With the improvement in standards of living and changing diets, the demand for quality edible oil is increasing. Thus, refining crude soybean oil is a necessary step in the production of soybean oils. Large amounts of high-strength organic wastewater are released during the crude soybean oil refining process, which usually includes degumming, deacidification, neutralization, bleaching, and deodorization steps to remove the undesirable components before making the oil available for human consumption [1]. The refined soybean oil wastewater (RSOW) has a high concentration of chemical oxygen demand (COD) and contains large amounts of sodium salts from free fatty acids soap stocks, oil, grease, sulfates, and phosphates [2]. The harmful effluent discarded in its raw form causes substantial impacts on the environment. RSOW is usually treated by a combination of a pretreatment to dislodge the oil and grease, biological treatment, and advanced treatment, and the removal of COD and oil content can reach more than 90% [3, 4]. However, the traditional treatment methods lack economic competition due to the increase in cost and energy. Because of the high concentration of organic materials, RSOW can be further used as a resource. Therefore, the development of an efficient and economical treatment approach for such RSOW is attractive.

The rapid decrease in the global reserves of fossil fuels, high energy prices, and concerns about environmental security has led to an increasing demand for research on renewable biofuels [5]. Single cell oils produced by oleaginous microorganisms are currently being researched as an alternative to plant-based oils for biodiesel synthesis due to their special characteristics, such as their abundance of sources, high lipid content, and short periods for production and immunity to the effects of season and climate [6, 7]. Oleaginous yeasts have attracted considerable interest because they quickly grow to high densities on a variety of carbon sources, and their large-scale culturing is easy to achieve. However, the cost of biodiesel is currently more expensive than that of conventional diesel due to the high cost share (70–85%) of the raw material [8, 9]. Increasing interest has been generated to explore ways to reduce the high cost of biodiesel, especially the cost of raw materials.

Industrial and agricultural wastewater and waste contain many organic compounds that can be utilized as low-cost raw materials for microbial oil production. Previous studies have been conducted using distillery and domestic mixed wastewater, olive mill wastewater, glutamate wastewater, and waste glycerol as substrates for microbial oil production by *Rhodotorula toruloides, Yarrowia lipolytica, Lipomyces starkeyi*, and *Trichosporon fermentans* [10–13]. However, both the biomass and lipid content were very low in the raw wastewater and waste due to insufficient amounts of phosphorus and carbon as well as a high concentration of ammonium-N (NH_4_^+^-N), which boosts cell growth but inhibits lipid synthesis [14]. It was noted that most of the wastewater and fermentation waste was mixed with additional nutrients, such as waste molasses and waste syrup [15]. RSOW contains an abundance of carbon sources and phospholipids and a low nitrogen content, which would provide a suitable environment for the growth and lipid accumulation of oleaginous yeast. To date, no work has used oleaginous yeasts to treat RSOW. *T. fermentans*, also known as *Dipodascus fermentans,* metabolizes many types of carbon sources. In a previous report, *Trichosporon* sp. grew in oil substrates, and the soybean oil promoted the production of lipids and intracellular lipases by *T. fermentans* [16, 17]. Hence, *T. fermentans* has the potential ability to metabolize the oil in RSOW. The aim of this study was to use *T. fermentans* to reduce the COD by converting the organic compounds in RSOW into lipids without sterilization, dilution, or nutrient supplementation, which would solve both an environmental problem and allow low-cost lipid production.

## Results and discussion

### The substrate metabolism and lipid accumulation of *T. fermentans* in RSOW

First, the original wastewater from the soybean oil refining plant was treated by *T. fermentans* without sterilization, dilution or nutrient supplementation. The removals of COD and oil content were 82.22% and 75.89%, respectively. It was proven that *T. fermentans* could grow properly in RSOW, and the effect of the treatment was satisfactory. However, the original wastewater was not suitable for studying substrate metabolism and lipid accumulation because of its inconsistent composition from different production phases or seasons and its corruptibility. The growth trend and wastewater treatment effect of *T. fermentans* on the simulated wastewater was similar to those on the original wastewater. Thus, the simulated RSOW was used for the next experiment based on its constant and controllable composition.

**Fig. 1:**
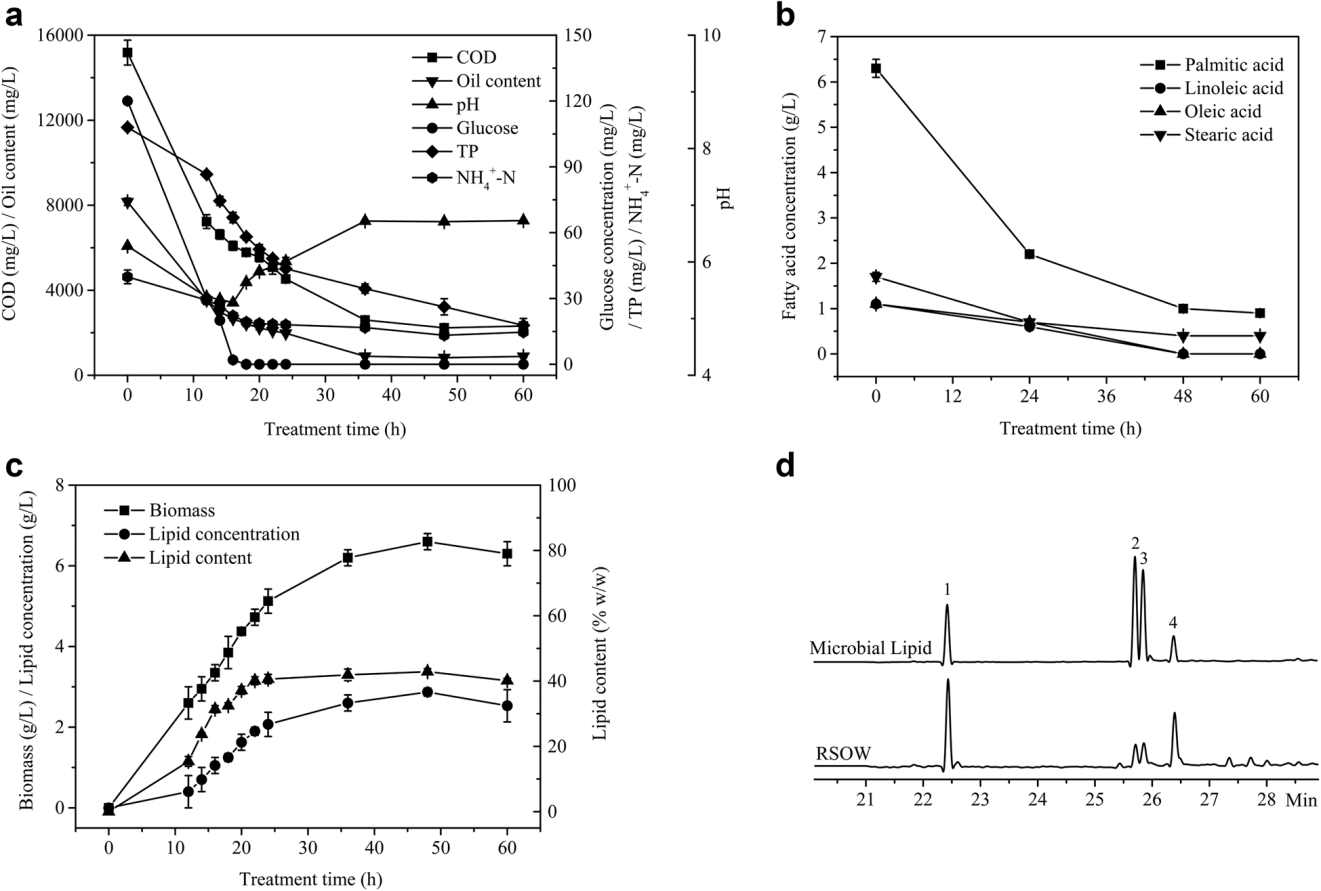
COD, oil content, TP and NH_4_^+^-N removals, glucose consumption, pH variation (**a**), palmitic acid, linoleic acid, oleic acid, and stearic acid consumption (**b**), biomass, lipid concentration, and lipid content (**c**), and GC analysis of the fatty acids including palmitic acid (1), linoleic acid (2), oleic acid (3), and stearic acid (4) in the RSOW and microbial lipids (**d**) of *T. fermentans* cultivation in the RSOW. The data represent the mean ± SD for triplicate samples

The major organic composition of RSOW includes glucose from the liquid portion of the seed, as well as soybean phospholipids, triglycerides, and sodium soap, which contribute to the oil content. The sodium soap was hydrolyzed to free fatty acids after adjusting the pH of the wastewater to 6 [18]. Thus, substrate metabolism was studied by analyzing the changing trends in the COD and oil, glucose, and fatty acid contents during wastewater treatment. As shown in Fig. 1a, the COD and oil content decreased rapidly from 0 to 24 h, and then the degradation rate gradually decreased to the lowest level at 48 h. The pH decreased with the rapid depletion of glucose from 0 to 18 h and then increased slowly. This result is probably because *T. fermentans* consumed a large amount of glucose for growth in the first 18 h [19]. Afterward, when *T. fermentans* no longer produced acids due to the depletion of glucose and accompanied by the expenditure of fatty acids, the pH increased. The same phenomenon was observed in acetone–butanol–ethanol fermentation wastewater (ABE), where the pH increased with acetic acid consumption [20]. Lv et al. [21] found that some yeasts had an absorptive effect on oily substances in wastewater. Therefore, the COD and oil content, which decreased significantly in the early stages of wastewater treatment, should be attributed to the absorption on the surface of *T. fermentans* to some extent. However, when the residual oil adsorbed on the surface of the yeast was stripped with a hot buffer after the treatment process was completed, it was found that the amount of adsorbed oil only accounted for less than 0.5% of the oil removed from the wastewater. This process showed that almost all of the removed oils, including those absorbed previously, had been used by *T. fermentans* for growth and lipid production during the process of wastewater treatment.

The composition and changes in fatty acids in the RSOW were analyzed (Fig. 1b). The RSOW mainly contained palmitic acid, oleic acid, linoleic acid, and stearic acid, and the unsaturated fatty acids (UFAS) amounted to approximately 22.3%. The four fatty acids were consumed simultaneously; linoleic acid and oleic acid were depleted at 48 h, but the consumption of palmitic acid and stearic acid rather than the other two fatty acids contributed more to the removal of the oil content during the treatment of the wastewater.

The time course of the biomass growth, lipid concentration, and lipid content by *T. fermentans* in the RSOW was investigated as shown in Fig. 1c. The biomass curve did not show three obvious growth stages, which may be attributed to the high inoculum concentration, the oxygen limitation in the culture, and that RSOW was different from the general culture medium. First, a rapid increase in the biomass was observed. Initially, the lipid content increased slightly and then increased sharply and reached a maximum. Second, from 24 to 48 h, *T. fermentans* accumulated many lipids. Finally, the biomass, lipid content and lipid concentration began to decrease. When the wastewater was treated for 48 h, the treatment efficiency was the best. The biomass and lipid content of *T. fermentans* was 6.6 g/L and 42.9% (w/w), respectively. The removals of the COD and oil content were 86.9% and 90.33%, respectively. The removals of total phosphorus (TP) and NH_4_^+^-N were 89.7% and 82.3%, respectively. In contrast, no microbial reproduction and no significant differences in the COD and oil content were detected in the uninoculated RSOW used under the same conditions, indicating that the *T. fermentans* treatment of RSOW achieved the dual purpose of effectively treating the wastewater and producing an abundance of microbial lipids.

The microbial lipids were composed of more than 80% triglycerides, and the composition of the fatty acids was of great concern, whether it was used as a raw material for biodiesel or the production of a special product with high added value [22, 23]. The fatty acid compositions of RSOW and lipids produced by *T. fermentans* in RSOW at 48 h were compared (Fig. 1d). Similar to the fatty acids in the wastewater, the microbial lipids also included palmitic acid, linoleic acid, oleic acid, and stearic acid. However, the wastewater was dominated by saturated fatty acids, while the microbial lipid content was mainly dominated by UFAS. In the process of wastewater treatment, a large amount of saturated fatty acids was bioconverted into UFAS by *T. fermentans*. The fatty acid composition of the microbial lipids was similar to that of vegetable oil, which is commonly used in biodiesel production. Thus, microbial lipids can be used as a potential raw material for biodiesel production [24].

The fatty acid composition of the microbial lipids produced by *T. fermentans* in the RSOW was also compared with those produced by *T. fermentans* in other cheap raw materials in other studies (Table 1). The lipids in the other studies were primarily composed of oleic acid, linoleic acid, palmitic acid, and stearic acid, but the content of UFAS was higher in this study. *T. fermentans* tends to synthesize microbial lipids with unsaturated fatty acids, such as oleic acid and linoleic acid, while utilizing different substrates as carbon sources. The synthesis of lipids by oleaginous yeast can be divided into two patterns: de novo lipogenesis, in which the lipids are accumulated and stored in cells through a series of physiological and biochemical processes when hydrophilic substrates such as glucose are used as carbon sources, and ex novo biosynthesis, which refers to the hydrophobic substrates, such as alkanes and fatty acids, from the environment that are transported into the cell and stored in the form of lipids [30]. The main organic compounds in the RSOW were sodium soap, phosphatides, and triglycerides composed of long chain fatty acids; thus, *T. fermentans* tended to accumulate lipids through ex novo biosynthesis in the RSOW. When the oil in the RSOW was utilized as carbon source by *T. fermentans,* its metabolic pathway is summarized as follows. First, the oil taken in by yeast cells was hydrolyzed to fatty acids under the action of lipase or phospholipase and then catalyzed by desaturase to produce more UFAS. The resultant fatty acids were activated into acyl-CoA, and phospholipids or triglycerides were synthesized by acyltransferase. Finally, lipid droplets were formed [31].

**Table 1:** Fatty acid composition of the lipids from *T. fermentans* in RSOW and other substrates

Substrate	Fatty acid composition of the lipids (%)						References
	Palmitic acid C16:0	Linoleic acid C18:2	Oleic acid C18:1	Stearic acid C18:0	Others	UFAS	
Sweet potato vines hydrolysate	32.5	20.5	22.3	22.7	2.0	42.8	[25]
Rice straw hydrolysate	24.6	10.5	53.2	11.7	–	68.2	[26]
Bagasse hydrolysate	27.5	10.1	54.2	5.8	2.4	64.3	[27]
Glycerol	27.5	10.1	54.2	5.8	2.4	60.0	[28]
Molasses	25.4	22.4	31.4	18.0	2.8	53.8	[29]
RSOW	19.6	36.4	35.5	8.5	–	71.9	This work

### Effect of the phospholipids in the RSOW on the treatment efficiency and lipid accumulation of *T. fermentans*

**Fig. 2:**
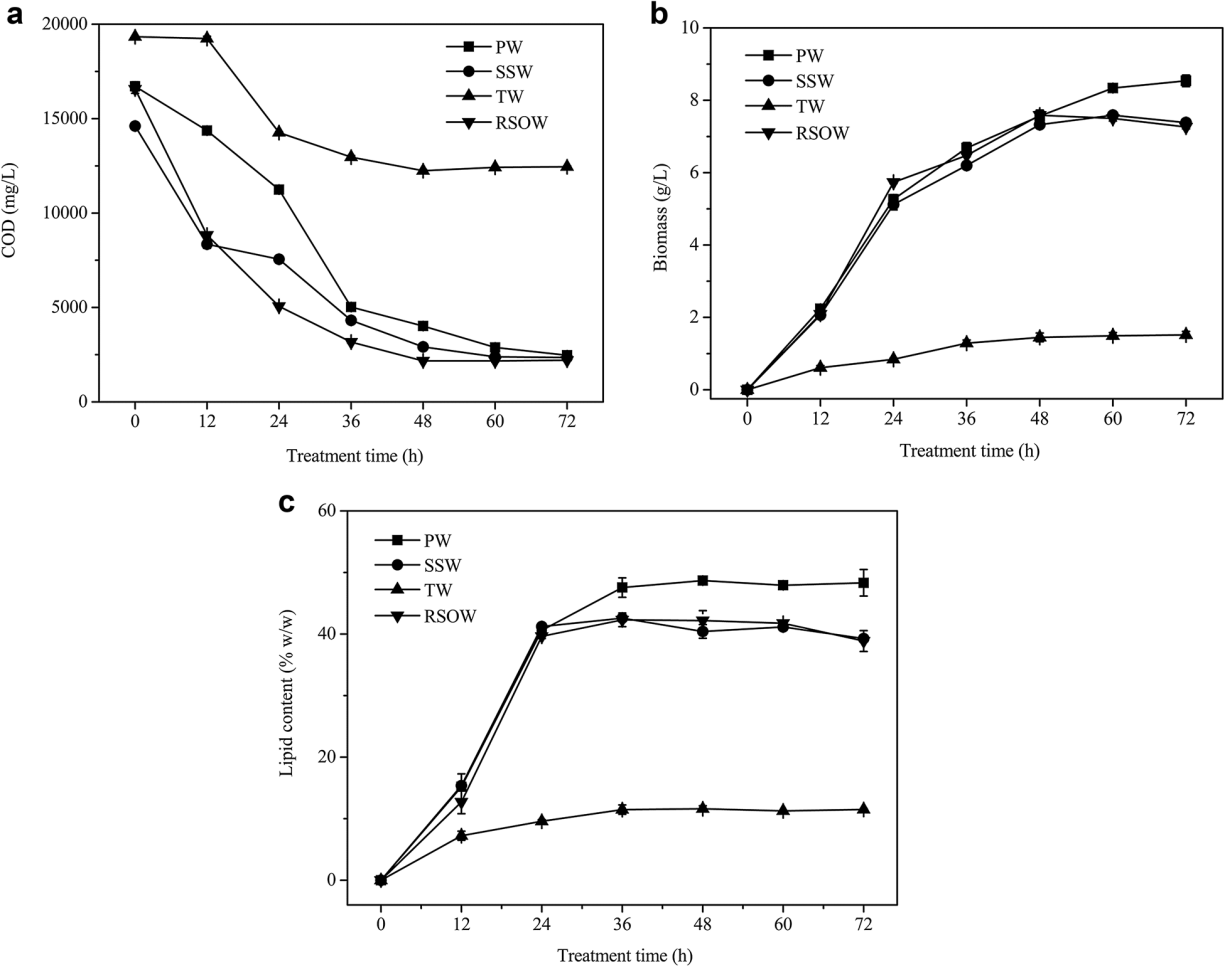
COD (**a**), biomass (**b**), and lipid content (**c**) of *T. fermentans* cultivation in PW, SSW, TW, and RSOW, respectively. The data represent the mean ± SD for triplicate samples

The oils in the RSOW were composed of phospholipids, sodium soaps, and triglycerides, but the composition of the oils in the RSOW from different sampled batches was unstable. A high concentration of PO_4_^3−^ may affect lipid accumulation by oleaginous yeasts in nitrogen-limited medium [32]. Therefore, to examine the impact of the phospholipids in the RSOW on the treatment efficiency and lipid accumulation of *T. fermentans*, the COD removal, biomass, and lipid content of wastewater that was prepared with 10 g/L phospholipids (PW), were tested under the same treatment conditions as the RSOW treated by *T. fermentans*. The results illustrated that a higher biomass and lipid content were obtained when phospholipids were the sole carbon source based on the fact that the phospholipids had no influence on the COD removal (Fig. 2). It is possible that the soybean phospholipids contained a small amount of inositol, which could promote the accumulation of lipids, and that resultant wastewater had an appropriate proportion of nutrients for lipid production [33]. Additionally, the higher concentration of phospholipids may have been transformed into more 3-phosphate-glycerol, which is a precursor of intracellular lipid synthesis. Wastewater with sodium soap (SSW) or triglycerides (TW) as the sole carbon source was also treated by *T. fermentans* (Fig. 2). In comparison with the COD removal, biomass, and lipid content of the RSOW, similar results were obtained in the SSW. In contrast, the results were inferior in the TW. Both the low solubility of triglycerides in water and the addition of surfactants to the TW to improve the solubility may have affected the cell growth and lipid accumulation.

### Optimization of the COD removal by Plackett–Burman design (PB) and Box–Behnken design (BBD)

**Fig. 3:**
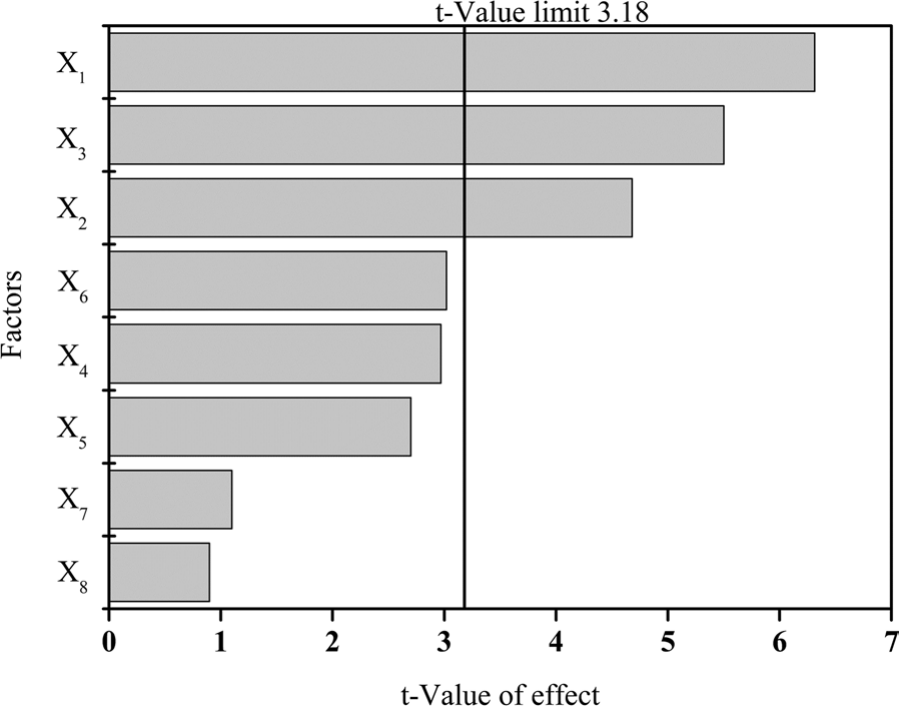
Pareto charts obtained after the PB experiment, showing the contribution of factors v/s their main effect on the COD removal

*Trichosporon fermentans* can degrade the oil and grease in wastewater and complete the pretreatment and biochemical treatment simultaneously, but the removal of COD did not attain the expected performance. In recent years, PB and response surface methodology (RSM) have been widely used to optimize microbial fermentation conditions and wastewater treatment parameters [34, 35]. In this study, the effects of the wastewater treatment were optimized by PB and RSM with the COD removal as the research objective. The temperature, initial pH value, inoculum concentration, shaker speed during cultivation, and initial substrate concentration are the main factors affecting the biological treatment of organic wastewater [36, 37]. Some metal ions can affect the growth morphology, the intra and extracellular osmotic pressure, and the activity of the key enzymes in the oleaginous yeasts during lipid accumulation [29]. It was revealed that 1.0 g/L MgSO_4_·7H_2_O, 2.0 mg/L ZnSO_4_·7H_2_O, and 0.3 mg/L CuSO_4_·5H_2_O benefited lipid production by *T. fermentans* [38]. Therefore, based on the above conditions, the variables that impacted the removal of the COD of the RSOW treated with *T. fermentans* were determined by PB. The Pareto chart describes the effect of the different variables on the COD removal (Fig. 3). The results showed that the order of influence of the eight variables on the COD removal was temperature (*X*_1_) > inoculum concentration (*X*_3_) > initial pH value (*X*_2_) > MgSO_4_ (X_6_) > shaker speed (*X*_4_) > initial COD concentration (*X*_5_) > ZnSO_4_ (*X*_7_) > CuSO_4_ (*X*_8_). Among them, the temperature, inoculum concentration, and initial pH value had significant effects on the COD removal and were tested by BBD.

The obtained data were used for a multiple regression analysis using Design-Expert 8.0.6 software, and the second-order polynomial equation of the COD removal (*Y*) for the wastewater treatment conditions, which were temperature (*X*_1_), initial pH value (*X*_2_), and inoculum concentration (*X*_3_), was fitted as follows:

1 $$\begin{aligned} Y \, = & - 142.55 + 2.61X_{1} + 62.88X_{2} + 3.14X_{3} \\ - 0.01X_{1} X_{2} + 0.07X_{1} X_{3} + 0.32X_{2} X_{3} \\ - 0.05X_{1}^{2} - 5.26X_{2}^{2} - 0.6X_{3}^{2}. \\ \end{aligned}$$

Analysis of variance (ANOVA) was used to evaluate the significance of the quadratic polynomial model (Table 2) [34]. The significance of every coefficient was examined with *P* values, which indicate the interaction strength of each parameter. It was observed that the model was highly significant at the 5% confidence level since the *P* values were less than 0.05. The lack-of-fit value was not significant (*P* = 0.1483), indicating that the equation was adequate for predicting the COD removal under different conditions [27]. The *R*^2^ value (0.9983) and adj-*R*^2^ value (0.9962) suggested good agreement between the experimental and predicted values and showed that 99.62% of the response variation was related to the variation in the independent variables [39]. All these considerations showed that the model was reliable for the prediction of the COD removal in this work and was suitable to fit the relationship between the response and the three independent variables.

**Table 2:** ANOVA for the COD removal according to the response surface quadratic model

Source	DF	Sum of squares	Mean square	*F* value	*P* value	
Model	9	0.49	0.054	469.25	< 0.0001	Significant
*X*_1_	1	1.469E−003	1.469E−003	12.93	0.0088	
*X*_2_	1	0.29	0.29	2487.42	< 0.0001	
*X*_3_	1	3.947E−003	3.947E−003	34.13	0.0006	
*X*_1_ *X*_2_	1	5.510E−006	5.510E−006	0.048	0.8334	
*X*_1_ *X*_3_	1	1.854E−004	1.854E−004	1.60	0.2460	
*X*_2_ *X*_3_	1	6.487E−004	6.487E−004	5.61	0.0497	
*X*_1_^2^	1	7.089E−004	7.089E−004	6.13	0.0425	
*X*_2_^2^	1	0.19	0.19	1612.25	< 0.0001	
*X*_3_^2^	1	2.391E−003	2.391E−003	20.68	0.0026	
Residual	7	8.097E−004	1.157E−004			
Lack of fit	3	5.689E−004	1.896E−004	3.15	0.1483	Not significant
Pure error	4	2.407E−004	6.018E−005			
Cor total	16	0.49				

*R*^2^ = 0.9983, Adj. *R*^2^ = 0.9962, Pred*R*^2^ = 0.9806, CV = 1.36%

**Fig. 4:**
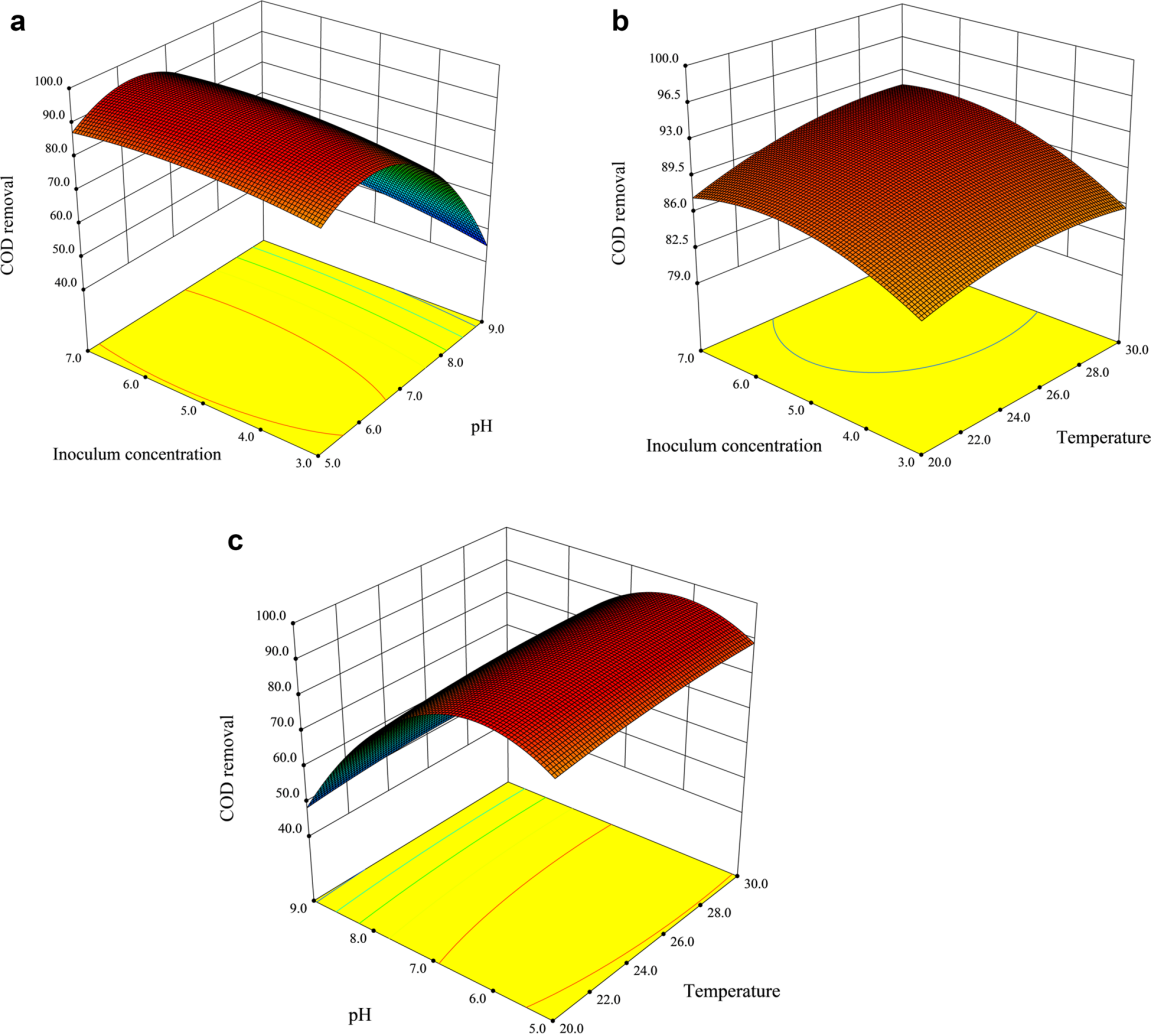
Response surface plots that show the binary interactions of different variables. The interactions between the inoculum concentration and initial pH (**a**), inoculum concentration and temperature (**b**), initial pH and temperature (**c**)

To further explore the interactions among the significant variables and determine the optimal levels, response surface curves of the above regression equation were plotted (Fig. 4). As shown in the surface plots, there were effective interactions between the inoculum concentration and initial pH (*P* = 0.0497). The interactions between temperature and each of the other two variables were not effective, indicating that temperature had little influence on the other two variables. According to the model, the optimal treatment conditions were obtained as follows: temperature 28.3 °C, initial pH value 6.1, and inoculum concentration 5.9% (v/v). *T. fermentans* was used to treat the RSOW under these conditions to verify the reliability of the RSM method. The COD removal was 92.1% and was very similar to the predicted value, which demonstrated that the optimal wastewater treatment conditions obtained by the RSM were reliable. In addition, the biomass was 7.5 g/L, the lipid concentration was 3.2 g/L, the lipid productivity was 0.07 g/(L·D), and the TP removal and the NH_4_^+^-N removal were 90.2% and 85.7%, respectively. The COD removal was increased by 7.1% using BBD, and the biomass and oil production were increased by 13.0% and 10.2%, respectively, in comparison to those before optimization (6.6 g/L and 2.9 g/L).

### RSOW treatment and lipid production in a 5-L bioreactor

**Fig. 5:**
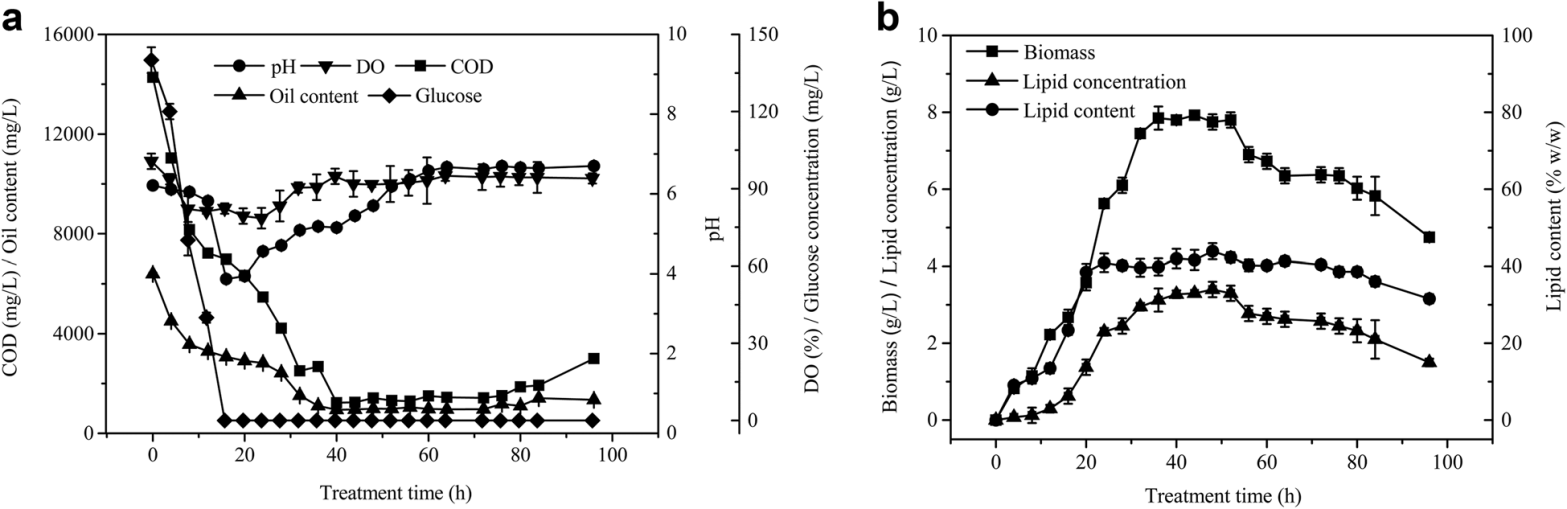
COD and oil content removal, glucose consumption, pH, and DO variation (**a**), biomass, lipid concentration, and lipid content (**b**) of *T. fermentans* cultivation in a 5-L bioreactor with RSOW. The data represent the mean ± SD for triplicate samples

The treatment of wastewater by microorganisms and the production of high value-added products are usually carried out in bioreactors, which can control environmental factors, such as temperature, pH, gas partial pressure, and nutrients, to meet the requirements of microorganismal growth and production [40]. The possibility of industrial applications can also be evaluated through bioreactor simulation experiments. To evaluate the feasibility of treating RSOW with *T. fermentans* on a large scale, the wastewater treatment was carried out in a 5-L bioreactor under the BBD-optimized conditions, which were temperature 28.3 °C, initial pH value 6.1, and inoculum concentration 5.9% (v/v). The bioreactor operated with the rotational speed of the mixing paddle and the air flow set at 400 rpm and 2 L/min, respectively. As shown in Fig. 5, the dissolved oxygen (DO) content decreased sharply with the rapid increase in biomass and lipid content from 0 to 24 h. The biomass growth decreased, and the lipid content remained stable, which led to a decrease in oxygen consumption and an increase in DO from 24 to 36 h. The growth of the oleaginous yeasts was in the stationary phase, and the DO remained the same starting at 36 h. The biomass and lipid content started to decrease, and the oleaginous yeast entered the death phase, causing the DO to increase again after 52 h. The changes in pH were the same as those of the flask experiment, which decreased early and increased later in the experiment. When the RSOW was treated with *T. fermentans* for 40 h, the COD and oil content decreased to an all-time low, and their removals were 94.7% and 89.9%, respectively. The biomass, lipid concentration, and lipid content reached a maximum, which were 7.9 g/L, 3.4 g/L, and 43% (w/w), respectively. Meanwhile, the removals of TP and NH_4_^+^-N were 90.4% and 85.6%, respectively. Compared with the outcome of the flask experiment, the wastewater treatment efficiency, biomass and lipid concentration increased slightly, and the treatment period of the wastewater was shortened from 48 to 40 h, resulting in an increase in lipid productivity to 0.09 g/(L D) when the experiment was executed in a 5-L bioreactor. When ABE, whose initial COD concentration was similar to that of RSOW, was treated with *Trichosporon cutaneum* under the same conditions of no pretreatment and nutrient supplementation, only a 68% COD removal, 4.9 g/L biomass, and 14.7% (w/w) lipid content were obtained [40]. Xue et al. [41] obtained a 25-g/L biomass, 20% (w/w) lipid content, and 45% COD removal after treating starch wastewater with *R. glutinis*, yet it was necessary to dilute the wastewater and supplement it with a large amount of glucose. When Xue et al. further enlarged the fermentation scale from 5 to 300 L, the treatment period was cut short from 60 to 30–40 h on the premise of maintaining a high removal of the COD and oil content [41]. Therefore, with an expansion in scale, it was possible to further shorten the treatment period of wastewater and improve the efficiency of the treatment and lipid production, which can provide conditions for batches or continuous treatment of wastewater. This experiment was of great practical importance for the potential industrial application of *T. fermentans* to treat RSOW.

## Conclusion

*Trichosporon fermentans* can be efficiently used for RSOW treatment and lipid production without sterilization, dilution, or nutrient supplementation and can complete pretreatment and biochemical treatment simultaneously, allowing the utilization of RSOW. The optimization of the treatment parameters by PB and BBD resulted in a remarkable increase in the COD removal of the RSOW and in lipid production. When the optimized conditions were expanded to a 5-L bioreactor, the wastewater treatment and lipid production were more efficient. The lipids from *T. fermentans* had a similar fatty acid composition to that of vegetable oils; thus, *T. fermentans* shows promise for biodiesel production.

## Methods

### Refined soybean oil wastewater

The original wastewater was obtained from a soybean oil refinery plant in Jilin City, China. The RSOW used in this study was simulated wastewater, which was prepared based on the results of the analysis of the original wastewater. The simulated wastewater was a mixture of 6.25 g/L sodium soap, 1.25 g/L soybean phospholipids, 2 g/L soybean oil, and 0.3 g/L (NH_4_)_2_SO_4_. The major characteristics of the resultant simulated wastewater were as follows: 20,000 ± 2000 mg/L COD, 8000 ± 200 mg/L oil content, 100 ± 15 mg/LTP, 50 ± 5 mg/L NH_4_^+^-N, and pH 10.0 ± 0.5.

### Microorganism, medium, and wastewater treatment

*Trichosporon fermentans* (CICC 1368) was purchased from the China Center of Industrial Culture Collection. The seed cells of these oleaginous yeasts were cultured in an autoclaved medium consisting of 20 g/L glucose, 10 g/L peptone, and 10 g/L yeast extract. After the pH of the RSOW without an autoclaving treatment was adjusted to 6.0, every 10 mL of seed cells were inoculated into 100 mL of wastewater placed into a 500-mL conical flask, which meant that the inoculum concentration was 10% (v/v). The wastewater treatment was performed in a rotary shaker at 30 °C and 150 rpm. At a given point in time, the treatment ended. The resultant 100 mL wastewater was used for assaying the biomass, lipid concentration, lipid content, and wastewater parameters. The RSOW without inoculation as the control was used under the same conditions. Triplicate samples were analyzed to calculate the mean and standard deviation (SD).

### Optimization of the COD removal by PB and BBD

Plackett–Burman design was used to screen the best candidate factors. The most effective variables on the COD removal of the RSOW treated with *T. fermentans* were selected using PB. All the experiments were conducted on the basis of a matrix designed by Design-Expert 8.0.6 (Stat-Ease, Inc., Minneapolis, USA). The eight independent variables selected were temperature (*X*_1_), initial pH value (*X*_2_), inoculum concentration (*X*_3_), shaker speed (*X*_4_), initial COD concentration (*X*_5_), MgSO_4_ (*X*_6_), ZnSO_4_ (*X*_7_), and CuSO_4_ (*X*_8_), and three virtual factors (*X*) were designed (Table 3).

**Table 3:** PB experimental design and results with the COD removal as the response variable

Independent variables												Response
Run	*X*_1_ (°C)	*X*_2_	*X*	*X*_3_ (% v/v)	*X*_4_ (rpm)	*X*	*X*_5_ (mg/L)	*X*_6_ (g/L)	*X*	*X*_7_ (mg/L)	*X*_8_ (mg/L)	COD removal (%)
1	20	9	1	6	100	− 1	10,350	0	− 1	0	0	56.50
2	30	9	− 1	6	150	1	10,350	1.0	− 1	0	2.0	73.01
3	20	6	− 1	2	100	− 1	10,350	1.0	− 1	0.3	2.0	55.18
4	20	9	1	2	150	1	19,020	1.0	− 1	0.3	0	41.80
5	30	6	− 1	2	150	− 1	19,020	0	− 1	0	0	70.08
6	20	6	− 1	6	100	1	19,020	1.0	1	0	0	89.36
7	30	6	1	6	150	− 1	10,350	1.0	1	0.3	0	81.38
8	30	9	1	2	100	− 1	19,020	1.0	1	0	2.0	67.10
9	20	9	− 1	6	150	− 1	19,020	0	1	0.3	2.0	47.94
10	30	9	− 1	2	100	1	10,350	0	1	0.3	0	57.80
11	20	6	1	2	150	1	10,350	0	1	0	2.0	39.02
12	30	6	1	6	100	1	19,020	0	− 1	0.3	2.0	87.71

Response surface methodology is a statistical method that is used to optimize an operational condition with multiple variables and study the relationship between each independent variable and its response [42, 43]. BBD was used for RSM in the experimental design, which is well suited for determining the optimal conditions for multifactorial systems quickly and effectively [35]. According to the results of PB, the three independent variables chosen were the temperature (*X*_1_), initial pH value (*X*_2_), and inoculum concentration (*X*_3_). Table 4A presents the variables and the operating ranges covered in BBD. A total of 17 experiments were generated on the basis of BBD using the same software, where the different variables were set at three levels (− 1, 0, + 1) to determine the COD removal under the optimum operational conditions (Table 4B). The quadratic model that describes both the linear trend and interactions of the responses as well as the independent three variables, was as follows:

**Table 4:** BBD for the optimization of the COD removal of the RSOW treated with *T. fermentans*

A Coding and levels of experiment factors				
Factor	Symbol	Code level		
		− 1	0	1
Temperature (°C)	*X*_1_	20	25	30
Initial pH value	*X*_2_	5	7	9
Inoculum concentration (% v/v)	*X*_3_	2	5	8

B Box–Behnken design arrangement and responses				
Run	*X*_1_	*X*_2_	*X*_30_	COD removal (%)
1	− 1	1	0	49.01
2	0	0	0	90.12
3	0	− 1	1	87.16
4	1	0	1	92.49
5	0	0	0	90.26
6	0	0	0	91.57
7	0	− 1	− 1	86.71
8	1	0	− 1	85.23
9	0	1	− 1	45.45
10	0	1	1	50.99
11	− 1	− 1	0	85.92
12	0	0	0	91.83
13	− 1	0	1	88.05
14	− 1	0	− 1	83.52
15	0	0	0	91.24
16	1	− 1	0	88.54
17	1	1	0	51.17

2 $$Y \, = \, \beta_{0} + \, \sum \beta_{i} X_{i} + \, \beta_{ij} X_{i} X_{j} + \, \sum \beta_{ii} X_{i}^{ 2}$$

Statistical analysis of the model was performed to evaluate the ANOVA, and response surface curves were generated using Design-Expert 8.0.6.

### Wastewater treatment in a 5-L bioreactor

*Trichosporon fermentans* was used to treat the RSOW without sterilization, dilution, or nutrient supplementation in a 5-L bioreactor (BIOTECH-5GB, Shanghai Baoxing, China) with a working volume of 2 L. The temperature, initial pH value, and inoculum concentration were set according to the BBD optimum operational conditions. The agitation and air flow rate were maintained at 400 rpm and 2 L/min for 96 h, respectively, with online monitoring of the pH and DO. Samples of 25 mL were withdrawn at intervals of every 4 h for analysis. The RSOW without inoculation as the control was operated under the same conditions.

### Analytical methods

The biomass, lipid concentration, and lipid content were determined as described by Wu [44]. The biomass was the cell dry weight per liter wastewater (g/L); the lipid concentration was the amount of lipid extracted from the cells per liter wastewater (g/L); and the lipid content was the mass percentage of lipid to biomass (% w/w) [45]. NH_4_^+^-N and TP were assayed with the Nessler’s reagent spectrophotometry method and ammonium molybdate spectrophotometric method, respectively [46, 47]. The glucose concentration was determined using an SBA-40D glucose analyzer (Shandong Academy of Sciences, Jinan, China). The COD was quantified with a COD-571 COD monitor (Shanghai Rex Water Quality Analysis Expert, China). The oil content was measured by a JLBG-129+ infrared spectrometric oil meter (Jilin Northlight Technology Co., Ltd, Jilin, China) [48].

The analysis for the fatty acid concentration of the wastewater was conducted on a 2010A HPLC (Shimadzu, Japan) with an Inertsil ODS C18 column (4.6 mm × 150 mm × 5 µm). A mixture of water and acetonitrile (25/75, v/v) was used as the mobile phase at a flow rate of 1.0 mL/min and 35 °C. The sample volume was 20 µL and was detected at 214 nm. The fatty acid concentration was calculated based on standard curves of commercial samples.

The fatty acid composition analysis of the lipids was performed on a GCMS-QP2010 (Shimadzu, Japan). The operation parameters of the GC analysis were as follows: flame ionization detector 250 °C; Rtx-5Sil MS column (30 m × 0.32 mm × 0.25 µm); carrier gas: helium (99.999%); and flow rate 1.0 mL/min. The profile of the column temperature was as follows: increased from 90 °C to 265 °C at 5 °C/min and maintained for 5 min. The operation parameters of the MS analysis were as follows: ion bombardment source: electron impact ion source; ion source temperature: 200 °C; electron multiplier voltage: 70 eV; scanning system: full scan; and solvent delay: 2.0 min. The relative content of fatty acids was calculated by the area normalization method.

## Abbreviations

RSOW: refined soybean oil wastewater
COD: chemical oxygen demand
NH_4_^+^-N: ammonium-N
ABE: acetone–butanol–ethanol fermentation wastewater
UFAS: the unsaturated fatty acids
TP: total phosphorus
GC–MS: gas chromatography–mass spectrometer
PW: wastewater with phospholipids
SSW: wastewater with sodium soap
TW: wastewater with triglycerides
PB: Plackett–Burman design
BBD: Box–Behnken design
RSM: response surface methodology
ANOVA: analysis of variance
DO: dissolved oxygen
SD: standard deviation
